# Intra-wound vancomycin powder for the eradication of periprosthetic joint infection after debridement and implant exchange: experimental study in a rat model

**DOI:** 10.1186/s12866-021-02399-5

**Published:** 2021-12-07

**Authors:** Jian Wei, Kai Tong, Siqi Zhou, Hui Wang, Yinxian Wen, Liaobin Chen

**Affiliations:** 1 Department of Joint Orthopedics, Affiliated Liutie Central Hospital of Guangxi Medical University, Liuzhou, 545007 China; 2 Division of Joint Surgery and Sports Medicine, Department of Orthopedic Surgery, Zhongnan Hospital of Wuhan University, No.169, Donghu Road, Wuhan, 430071 China; 3 Department of pharmacology, Basic Medical School of Wuhan University, Wuhan, 430071 China

**Keywords:** Periprosthetic joint infection, One-stage exchange arthroplasty, Intra-wound vancomycin powder, Rat model

## Abstract

**Background:**

Intra-wound vancomycin powder (VP) has been used in clinical practice to prevent periprosthetic joint infection (PJI) after primary knee/hip arthroplasty. The role of intra-wound VP in the setting of debridement and implant exchange after PJI remains undefined. This study aimed to explore the efficacy and safety of intra-wound VP in the control of methicillin-resistant *S. aureus* (MRSA) infection after debridement and implant exchange.

**Methods:**

PJI modeling by knee prosthesis implantation and MRSA inoculation, debridement and implant exchange were performed in Wistar rats successively to mimic the one-stage exchange arthroplasty of PJI patients. Two weeks of systemic vancomycin (SV) or/and intraoperative intra-wound VP of single dosage were applied after revision surgery.

**Results:**

No post-surgery deaths, incision complications and signs of drug toxicity were observed. The microbial counts of SV or intra-wound VP group were significantly reduced compared with the control group, while bacteria were still detected on the bone, soft-tissue and prosthesis. The elimination of bacterial counts, along with improvement of tissue inflammation and serum inflammatory markers, were observed in the rats with SV plus intra-wound VP. Serum levels of vancomycin in all groups were lower than that of causing nephrotoxicity, while no statistic difference was observed in the serum biochemical marker among the groups.

**Conclusions:**

Intra-wound VP is effective after debridement and implant exchange in our current rat PJI model. Neither SV nor intra-wound VP alone could eradicate the bacteria within a two-weeks treatment course, while SV plus intra-wound VP could eliminate the MRSA infection, without notable hepatic or renal toxicity and any incision complications.

## Introduction

Periprosthetic joint infection (PJI) is one of the most common causes of revision total knee/hip arthroplasty, which counts for up to 25.2% of the revision arthroplasties [1, 2]. *S. aureus* is one of the most common pathogens for all the PJI cases [3], while approximately 47% of *S. aureus* clinically isolated in the United States are MRSA [4]. Currently study reported that after revision arthroplasty of MRSA infection, the risk of failure or re-infection was much higher than methicillin-sensitive *S. aureus* [3], of which the recurrence rate of infection was up to 28.6% [5–7].

One-stage exchange arthroplasty, namely immediate implant exchange after debridement, is one of the important strategies for PJI treatment, which is praised highly by orthopedic scholars in recent years. However, the infection eradication rate was still not satisfactory, approximately 5–25% of patients failed in eliminating the infection after one-stage exchange arthroplasty [8]. In addition to complete debridement, perioperative management of antibiotics is extremely important. The current recommendation by systemic vancomycin post-surgery as the preferred therapeutic antibiotics for PJI caused by MRSA may not be adequate. Generally, systemic vancomycin fails to reach the minimum biofilm eradication concentration (MBEC) of the synovial fluid and infected tissues [9, 10], while increasing the dosage, concentration and duration of systemic vancomycin may increase the risk of drug adverse reactions. Even if vancomycin-cemented prosthesis was used for some single-stage exchange surgeries, vancomycin in the cement-fixation should be limited to 1-2 g per 40 g cement powder, or the mechanical properties of antibiotic-cement may be significantly decreased [11]. In addition, in vitro studies suggested that less than 5% of total vancomycin in the cement were eventually released in two-months elution [12–14]. Obviously, systemic vancomycin and/or vancomycin-cemented prosthesis seems not the best solution for increasing the concentration of vancomycin in the synovial fluid and infected tissues around the joint. Thus, local application of antibiotics such as vancomycin attracts the attention of the surgeons currently. Some clinical studies had demonstrated that intra-wound VP could reduce the incidence of infections in the infection prophylaxis of primary knee/hip arthroplasty and spine surgery due to the high local concentration of vancomycin [15, 16]. However, several studies suggested that intra-wound VP did not alter the infection rate but increased the incidence of wound complications [17–21]. To our knowledge, the efficacy and safety of intra-wound VP in the one-stage exchange arthroplasty has not been evaluated in the previous researches.

Herein, this study intended to explore the efficacy and safety of intra-wound VP in the PJI controlling caused by MRSA after debridement and implant exchange in a rat model, to provide experimental basis for the clinical development of postoperative antibiotic management plan.

## Materials and methods

### Animals and reagents

Wistar rats of SPF grade (male, aged 11 weeks, weighted 285 g ± 6 g). All animal experimental procedures were performed following the Guidelines for the Care and Use of Laboratory Animals of the Animal Welfare Committee. Clinical-grade vancomycin hydrochloride for injection was obtained from Lilly (Japan). Dosages of vancomycin were based on the vancomycin therapeutic guidelines of human and used in prior PJI patients and rat models [15, 17, 22–26].

### Bacteria

Individual colonies (MRSA; ATCC 43300) were grown in Luria-Bertani (LB) broth. When log-phase growth was achieved, bacterial suspension was centrifuged and the supernatant was discarded, bacteria were resuspended with PBS solution to achieve a concentration of approximately 1.5 × 10^6^ CFU/ml as confirmed by serial dilution and plating on agar plates. In a pilot study, we established that 50 μl of 1.5 × 10^6^ CFU/ml ATCC 43300 inoculation was sufficient to reliably produce a PJI Wistar rats in 2 weeks. The minimal inhibitory concentration (MIC) of vancomycin in MRSA (ATCC-43300) was detected by microbroth dilution method. Vancomycin was added to 96-well plate with serial dilutions, the initial bacterial concentration in each well was adjusted to a concentration of 10^5^ CFU/ml and incubated at 37 °C for 24 h. The vancomycin MIC was defined as the lowest concentration that inhibited visible growth.

### Surgical procedure and study design

**Fig. 1:**
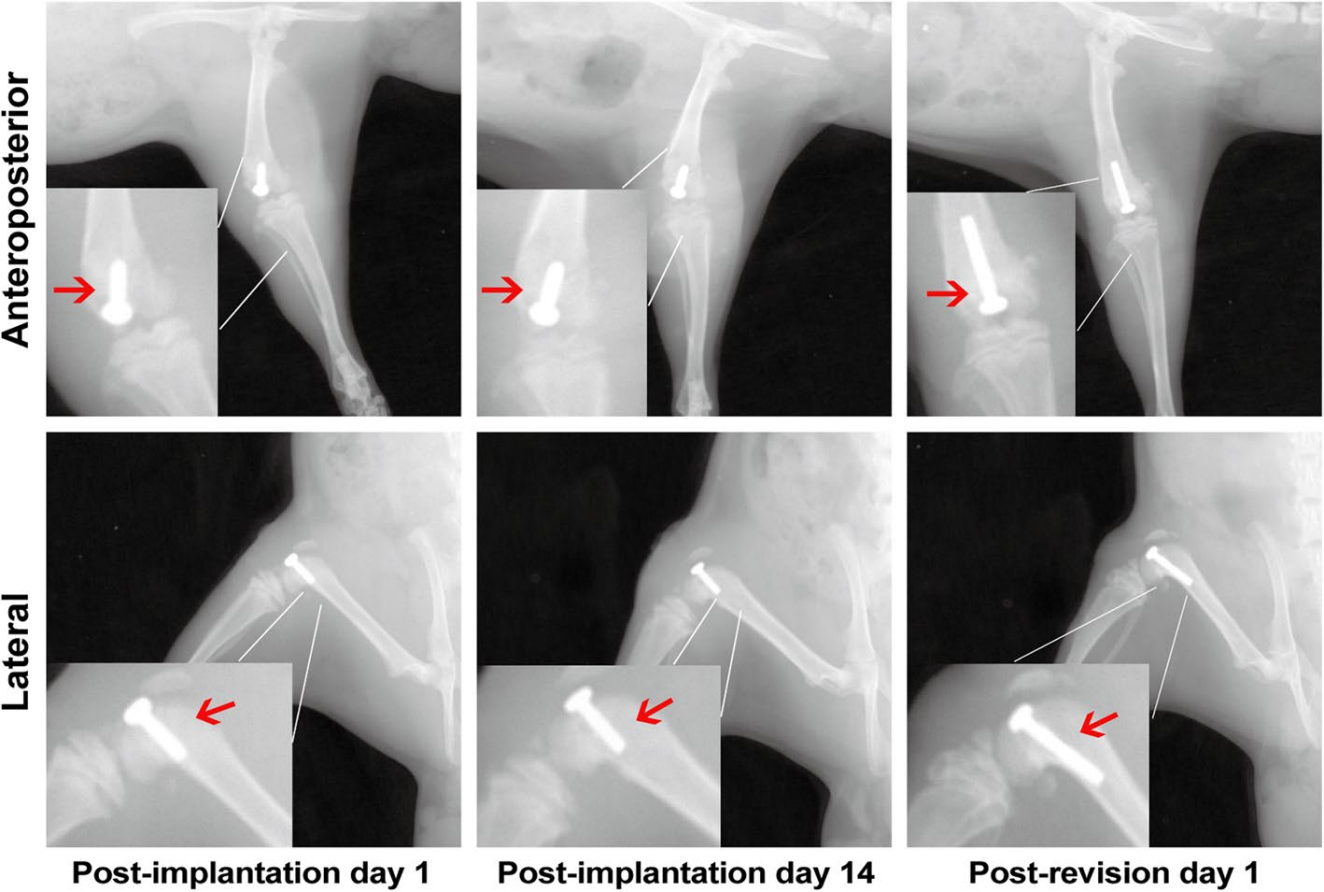
Radiological evaluation after knee prosthesis implantation (post-implantation day 1 and days 14) and one-stage exchange arthroplasty surgery (post-revision day 1). Anterior-posterior and lateral X-ray images were taken of right hind limbs of rats after knee prosthesis implantation and one-stage exchange arthroplasty to confirm the position of prosthesis and osteolysis around the prosthesis

Briefly, general anesthesia was induced by intraperitoneal administration of ketamine (60 mg/kg) and xylazine (6 mg/kg). After surgical anesthesia, the right legs of all rats were shaved, the skin was disinfected, the knee joint was surgically exposed, and a 1.3-mm hole was drilled into the femoral canal just anterior to the Blumensaat line. The prosthesis (diameter 1.5 mm, length 5 mm) was manually placed through retrograde insertion with a screwdriver, with 1 mm screw cap protruding into the joint (Fig. 1). After the capsule was sutured, 50 μl of 1.5 × 10^6^ CFU/ml suspension of ATCC 43300 was injected into the articular cavity. Pain was controlled with buprenorphine within 3 days post-surgery (0.1 mg/kg). On days 14 after surgery and bacterial inoculation, X-rays showed prosthesis loosening and osteolysis (Fig. 1). The prosthesis was removed by surgery aseptically, then infected and inflammatory synovium and soft tissues were removed, the femoral canal was cleaned and slightly reamed, soaking in dilute betadine lavage for 10 min, and washing with saline and dilute betadine repeatedly until intra-wound tissues were fresh. New elongated prosthesis (diameter 1.6 mm, length 8 mm) were implanted after debridement (Fig. 1). Sixty rats were randomly divided into four treatment groups after debridement and implant exchange: (1) Control (no antibiotics, *n* = 15), (2) systemic vancomycin (intraperitoneal injection, 88 mg/kg, every 12 h (q12 h), *n* = 15; equal to 1 g in a 70 kg patient, q12 h), (3) intra-wound VP [medullary cavity, articular cavity, prosthesis interface, synovial surface and intra-wound soft tissues were uniformly distributed] (before closure of the capsule, 88 mg/kg, *n* = 15; single dosage), and (4) systemic vancomycin & intra-wound VP (*n* = 15). Animals that assigned to systemic vancomycin were administered for 2 weeks. All animals were euthanized on days 28 for tissue harvest in accordance with the Institutional Animal Care and Use Committee approved protocol.

### Serum biochemical markers and serum levels of vancomycin

Serum samples were obtained by centrifugation (3000 rpm, 4 °C, 15 min). The serum alpha-1-acid glycoprotein (α1-AGP), creatinine (Cr), alanine aminotransferase (ALT), and aspartate aminotransferase (AST) were measured by ELISA kit (CUSABIO, China; as described in the figure legends). The serum levels of vancomycin at 0.5 h, 2 h, 4 h and 12 h after vancomycin first application were detected by high-performance liquid chromatography-mass spectrometry (HPLC-MS, Thermo TSQ Quantis, USA; filter: SRM MS2 725.80–1307.30 m/z; mass: 1307.30 m/z; retention time: 1.58 min; solvents: 1% formic acid water and pure acetonitrile; columns: Hypersil GOLD, Thermo Fisher, 100 × 2.1 mm, 3 μm; flow rates: 0.2 ml/min; time: 6 min).

### X-ray evaluation

Anterior-posterior and lateral X-ray images were taken from the right limbs to confirm the position of the prosthesis and the osteolysis around the prosthesis (Bruker, Germany; Filter: 0.4 mm, 45kvp, Exposure time: 1.2 s, Bin: 1 × 1 Pixels, FOV: 10 cm, fStop: 2).

### Scanning Electron microscopy of prosthesis

Prosthesis was carefully removed and the surfaces were examined by a single, experienced observer blinded to treatment. The samples were fixed (2.5% glutaraldehyde 4 °C 24 h, osmium acid 2 h), dehydrated in an alcohol gradient (concentration of 50, 60, 80, 95, and 100%, for 10 min per concentration), dried in an EM CPD300 Critical Point Dryer (Leica, Germany), coated with a conductive coating using a Q150R S Plus Sputter Coater (Quorumtech, England), and observed using Zeiss Auriga field emission scanning electron microscope (SEM) and Gatan digital camera system (Zeiss, Germany). MRSA was identified referring to the previous literature [27]. Five fields of view (FOV) were randomly selected from each specimen for observation under high magnification (5000×) and MRSA in each FOV were counted.

### Microbiological evaluation

On days 28, the surgical incision was reopened under sterile conditions. Sterile surgical instruments were used to harvest wound tissue, including all the muscles and soft tissue around the knee, knee joint bone, prosthesis. The tissues (bone and soft tissues) were combined with the same amount (8 ml) of sterile PBS solution, respectively, then homogenized with a fast tissue grinder (70HZ, 10 min; JXFSTPRP, China). Then, 100 μl of supernatant was inoculated onto LB agar Petri dishes and grown for 24 h at 37 °C. The retrieved prosthesis was placed in 2 ml of sterile PBS solution (containing 0.3% Tween 20) and sonicated to stimulate release of bacteria biofilm from the prosthesis. 100 μl of prosthesis supernatant was inoculated as prior descriptions [16, 28, 29]. Bacterial colonies were quantified using plate count method.

### Histopathological evaluation

Histologic analyses (knee joint) were carried out to assess the tissue morphology with particular attention toward signs of inflammation, bone necrosis and osteomyelitis. After decalcification [0.3 M EDTA, 28 days] (bone), dehydration (ethanol, xylene, and paraffin), and paraffin-embedding (all samples), samples were sectioned (4 μm) and stained with hematoxylin and eosin (H&E). All the slices were observed and photographed by H550S Photo Imaging System (Nikon, Japan), and assessed by an experienced observer blinded to treatment.

### Statistical analysis

Data were analyzed using SPSS software (version 22.0; SPSS Inc., USA) and are presented as the mean and standard error of the mean. Data were compared by two-way analysis of variance (ANOVA), or unpaired 1-tailed Mann-Whitney test (see figure legends). *P* values of < 0.05 were considered significant.

## Results

### Assessments of serum infection biomarker

**Fig. 2:**
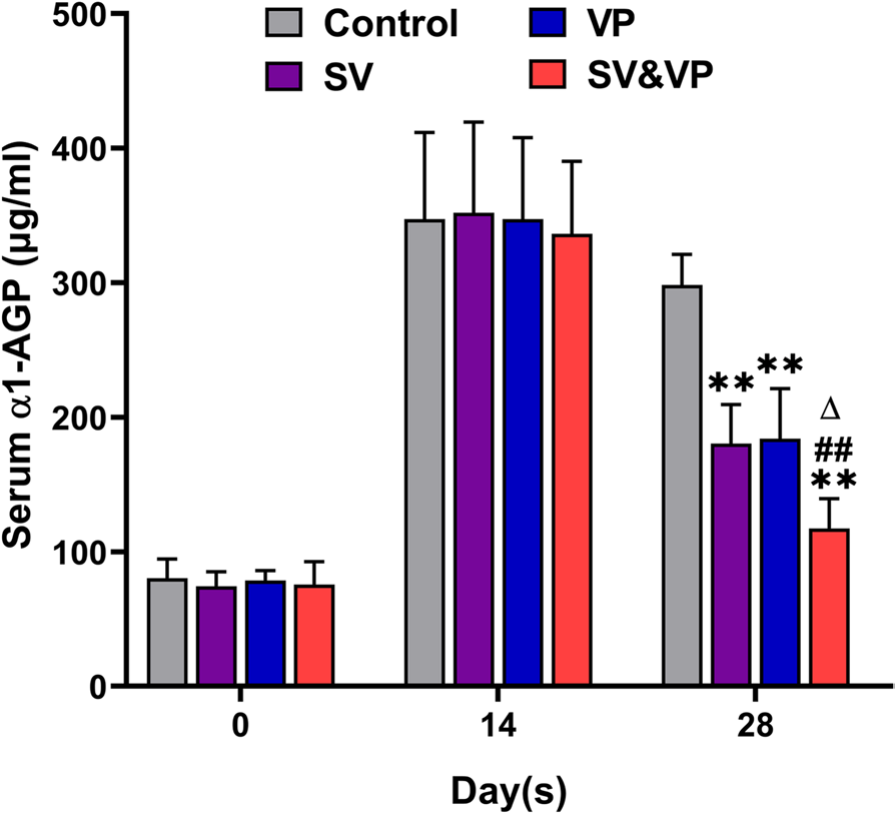
The serum α1-AGP during the whole experiment (pre-operation, days 14 and 28). Control (no antibiotics); SV: Systemic vancomycin (intraperitoneal injection, 88 mg/kg, every 12 h); VP: Intra-wound vancomycin powder (before closure of the capsule, 88 mg/kg, single dosage); SV&VP: Systemic vancomycin & intra-wound VP (intraperitoneal injection, 88 mg/kg, every 12 h; combined with intra-wound VP before closure of the capsule, 88 mg/kg, single dosage). **P < 0.01 (Compared with Control group), ^**##**^*P* < 0.01 (Compared with VP group), ^∆^*P* < 0.01 (Compared with SV group). *n* = 8

The α1-AGP is a characteristic serum biomarker of acute infection in rats. The mean values of α1-AGP in the Control, SV, VP and SV&VP groups were 80.31 ± 14.33 μg/ml, 74.38 ± 10.82 μg/ml, 78.74 ± 7.18 μg/ml, and 75.73 ± 16.88 μg/ ml before surgery. On days 14 after bacterial inoculation, serum α1-AGP of each group were 347.32 ± 64.43 μg/ml, 351.94 ± 67.48 μg/ml, 347.33 ± 60.44 μg/ml, and 336.31 ± 53.85 μg/ml, respectively, which were all significantly higher than those preoperative (Fig. 2, *P* < 0.01). On days 28, α1-AGP of the Control, SV, VP and SV&VP groups were 298.30 ± 22.71 μg/ml, 168.18 ± 26.92 μg/ml, 176.02 ± 38.16 μg/ml, and 117.37 ± 22.14 μg/ml, respectively, indicating that α1-AGP in the vancomycin treatment groups were significantly lower than the Control group (*P* < 0.01). No significantly differences were detected between the SV and VP group (*P* > 0.05), while the SV & VP group showed the lowest serum α1-AGP levels in all treatment groups (Fig. 2, *P* < 0.01).

### X-ray evaluation of the knee

**Fig. 3:**
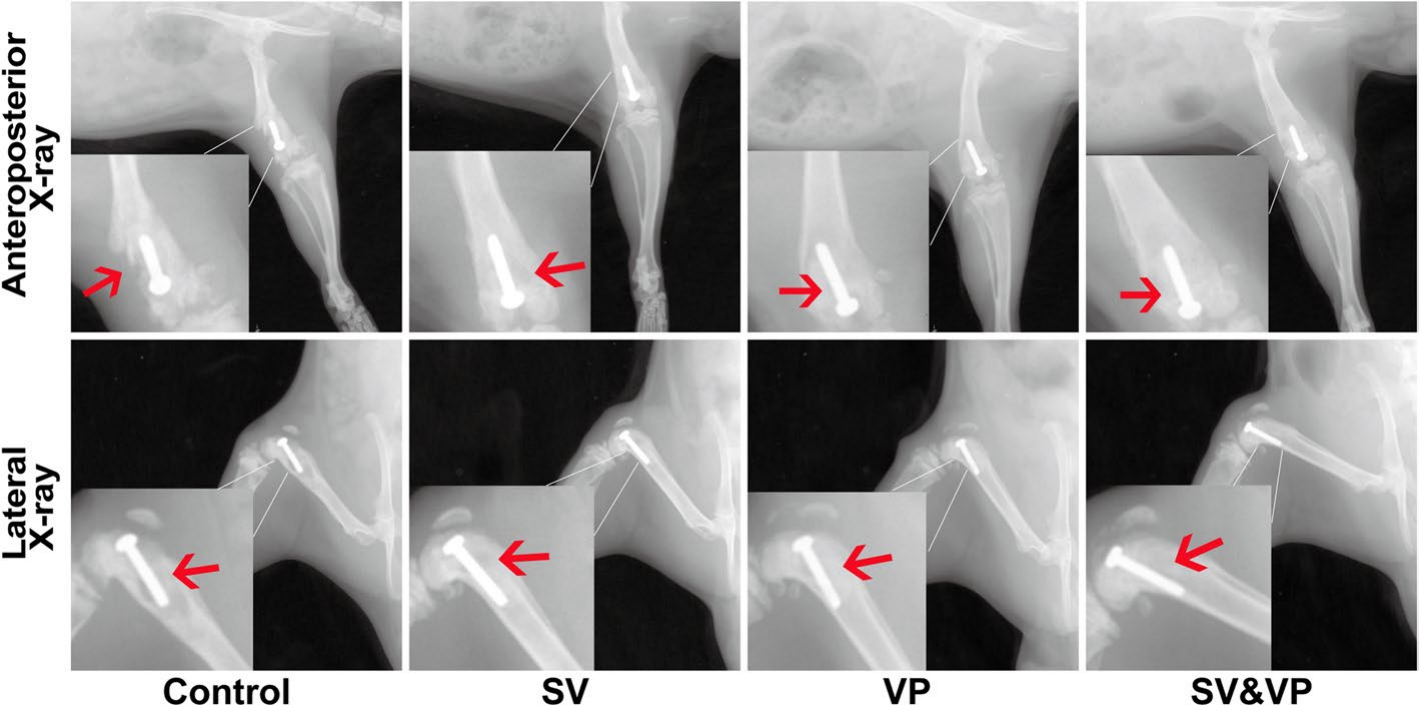
X-ray evaluation of the knee joint and prosthesis on days 28. Control (no antibiotics); SV: Systemic vancomycin (intraperitoneal injection, 88 mg/kg, every 12 h); VP: Intra-wound vancomycin powder (before closure of the capsule, 88 mg/kg, single dosage); SV&VP: Systemic vancomycin & intra-wound VP (intraperitoneal injection, 88 mg/kg, every 12 h; combined with intra-wound VP before closure of the capsule, 88 mg/kg, single dosage). The red arrow indicates the position of prosthesis and destruction of the bone

On days 14, signs of prosthesis loosening and osteolysis around the prosthesis were observed, indicating a local infection in the knee (Fig. 1). On days 28, prostheses were still in the position of distal femoral metaphysis, but all of them were accompanied by signs of prosthesis loosening and osteolysis around the prosthesis, of which the Control group was the most serious, while milder osteolysis was observed in the rats from SV, VP and SV & VP groups, especially in the SV & VP group (Fig. 3).

### Microbiological evaluation

**Fig. 4:**
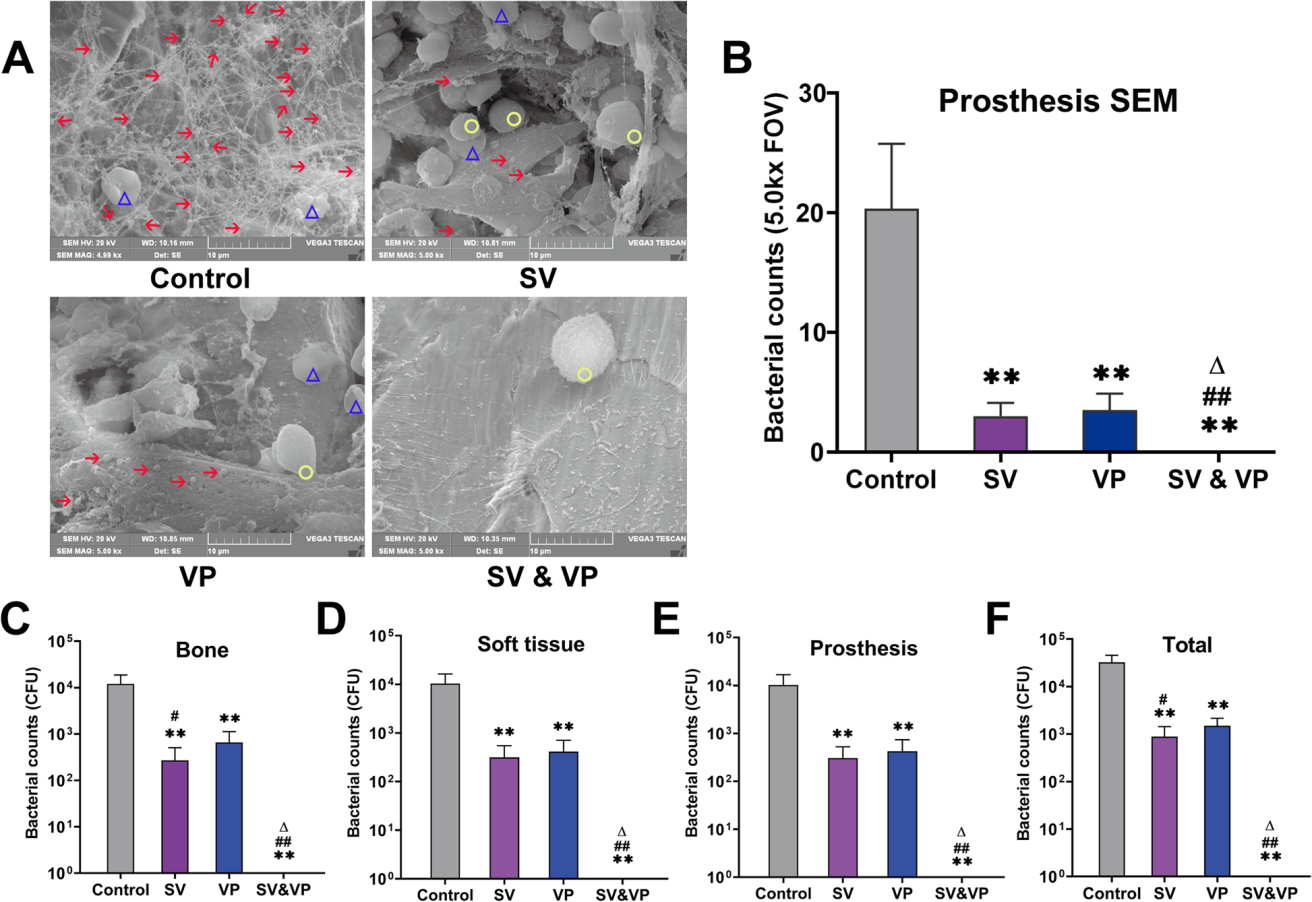
Microbiological evaluation in each treatment group on days 28. **A** The microbes on the surface of the prosthesis (*n* = 5) in each treatment group were observed by scan electron microscopy (SEM), with high magnification (5000×). **B** Bacteria counting by SEM. Five fields of view (FOV) were randomly selected from the prostheses for observation under high magnification (5000×) of SEM and bacterial counting. **C** The analysis of microbial culture counts of knee joint bones of animals in each treatment group. **D** The analysis of microbial culture counts of all soft tissues around the knee of animals in each treatment group. **E** The analysis of the microbial culture counts of the prosthesis of animals in each treatment group. **F** The analysis of microbial culture counts of the whole animal in each treatment group. Control (no antibiotics); SV: Systemic vancomycin (intraperitoneal injection, 88 mg/kg, every 12 h); VP: Intra-wound vancomycin powder (before closure of the capsule, 88 mg/kg, single dosage); SV&VP: Systemic vancomycin & intra-wound VP (intraperitoneal injection, 88 mg/kg, every 12 h; combined with intra-wound VP before closure of the capsule, 88 mg/kg, single dosage). **P < 0.01 (Compared with Control group), ^**#**^*P* < 0.05, ^**##**^*P* < 0.01 (Compared with VP group), ^∆^*P* < 0.01 (Compared with SV group). *n* = 10. The red arrow indicates methicillin-resistant *staphylococcus aureus* (MRSA), the yellow circle indicates leukocyte, and the blue triangle indicates erythrocyte

A greater quantity of MRSA particles was observed in the Control group, which was surrounded by host erythrocyte. No other microbial contamination was found in any field of view. Compared with the Control group, bacterial counts on the prostheses in the SV, VP and SV & VP groups were decreased, while no bacteria were observed in the SV & VP group (namely the bacteria on the surface of prosthesis were completely eliminated) (Fig. 4A-B). The bacterial colonies of each specimen and the whole animal in each treatment group were shown in Table 1 and Fig. 4C-F. The average CFUs of each specimen in vancomycin treatment groups were significantly less than the Control group, bacterial counts and whole animal in the SV group were less than the VP group, both of the SV and VP groups were higher than that of the SV & VP group, as no bacterial colonies were observed in any specimen in the SV & VP group (Fig. 4C-F, *P* < 0.01).

**Table 1:** Mean colony-forming units (CFUs) data from Fig. 4C–F

Group	Bone	Prosthesis	Soft tissue	Total
Control	1.19 × 10^4^ ± 6.76 × 10^3^	1.03 × 10^4^ ± 5.93 × 10^3^	1.02 × 10^4^ ± 6.51 × 10^3^	3.26 × 10^4^ ± 1.32 × 10^4^
SV	269 ± 237	313 ± 234	307 ± 219	889 ± 549
VP	660 ± 469	414 ± 292	427 ± 313	1501 ± 658
SV&VP	0	0	0	0

Control (no antibiotics); *SV* Systemic vancomycin (intraperitoneal injection, 88 mg/kg, every 12 h), *VP* Intra-wound vancomycin powder (before closure of the capsule, 88 mg/kg, single dosage), *SV&VP* Systemic vancomycin & intra-wound VP (intraperitoneal injection, 88 mg/kg, every 12 h; combined with intra-wound VP before closure of the capsule, 88 mg/kg, single dosage). *n* = 10

### Tissue inflammation evaluation

**Fig. 5:**
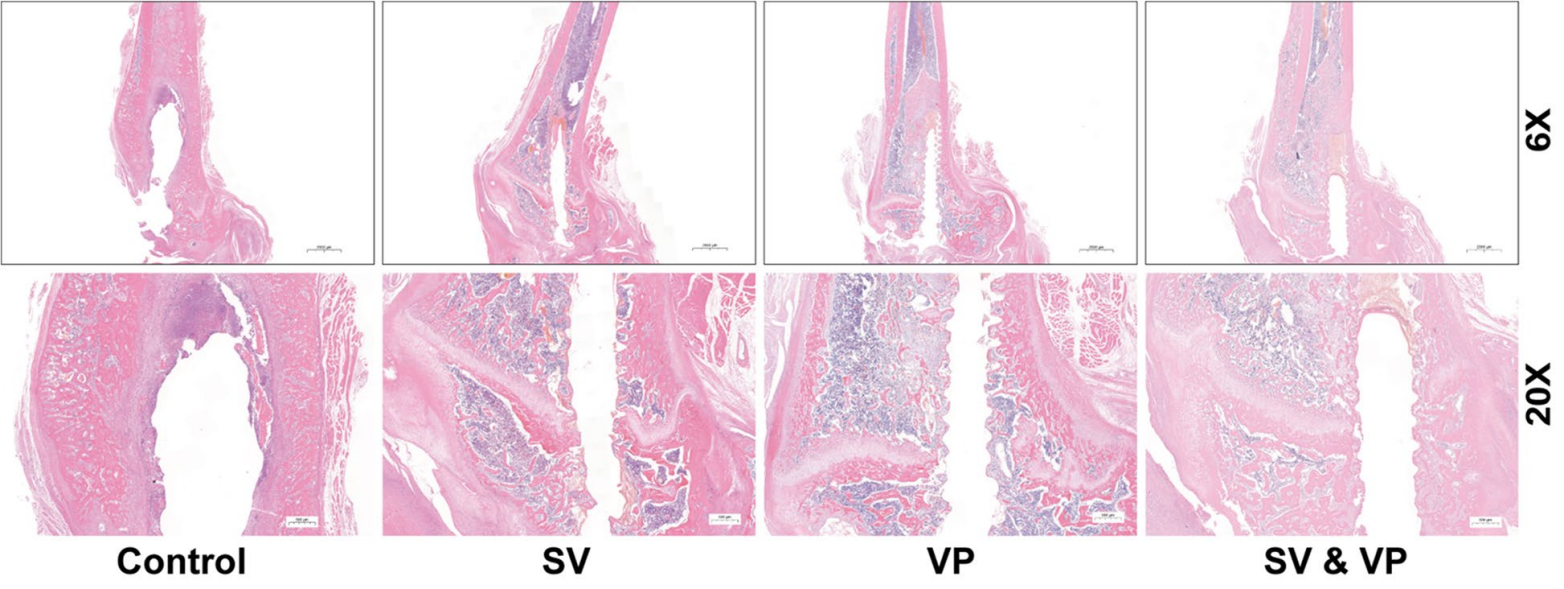
Histopathological assessment of the distal femur on days 28, with magnification (6× and 20×). Control (no antibiotics); SV: Systemic vancomycin (intraperitoneal injection, 88 mg/kg, every 12 h); VP: Intra-wound vancomycin powder (before closure of the capsule, 88 mg/kg, single dosage); SV&VP: Systemic vancomycin & intra-wound VP (intraperitoneal injection, 88 mg/kg, every 12 h; combined with intra-wound VP before closure of the capsule, 88 mg/kg, single dosage). *n* = 5

Osteomyelitis changes were observed in the Control animals, such as intramedullary abscess, necrotic bone formation, trabecular bone structure changes and inflammatory cell aggregation, while all these changes were attenuated after vancomycin treatment, especially in the SV&VP group. Almost no obvious inflammatory cells infiltration was observed in the SV&VP group (Fig. 5).

### Safety evaluation of intra-wound VP or/and systemic vancomycin in one-stage exchange arthroplasty

**Fig. 6:**
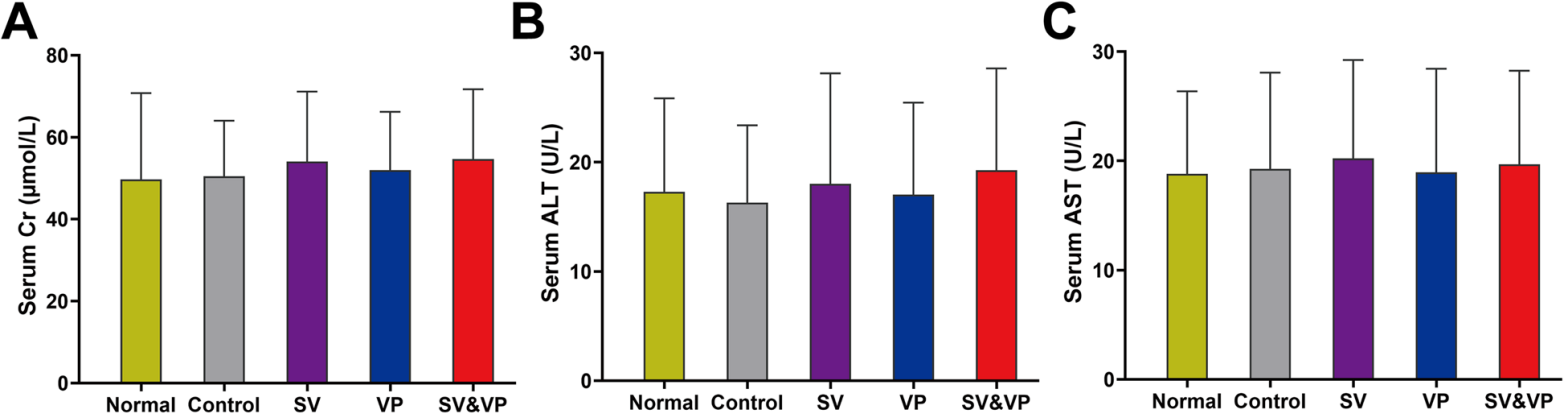
Measurement of Serum biochemical markers on days 28 (post-revision days 14). **A** Measurement of serum creatinine (Cr) on days 28. **B** Measurement of serum alanine aminotransferase (ALT) on days 28. **C** Measurement of serum aspartate aminotransferase (AST) on days 28. Normal (pre-operation); Control (no antibiotics); SV: Systemic vancomycin (intraperitoneal injection, 88 mg/kg, every 12 h); VP: Intra-wound vancomycin powder (before closure of the capsule, 88 mg/kg, single dosage); SV&VP: Systemic vancomycin & intra-wound VP (intraperitoneal injection, 88 mg/kg, every 12 h; combined with intra-wound VP before closure of the capsule, 88 mg/kg, single dosage). *n* = 15

Macroscopically, the incisions of each group were healed without wound rupture or exudation. The serum levels of vancomycin in the SV, VP and SV & VP groups were all higher than the MIC of MRSA (ATCC 43300; 2 μg/ml) at 0.5 h after vancomycin administration, but lower than the serum level that could cause nephrotoxicity (15–20 μg/ml) [30–33]. At post-surgery 12 h, the serum levels of the vancomycin treatment groups were lower than the limit of detection (Table 2). Moreover, the serum Cr, ALT and AST of each treatment group were all within the normal range, while no significant difference was observed among the four groups on days 28 and pre-operative values (Fig. 6, *P* > 0.05).

**Table 2:** Serum levels of vancomycin at 0.5, 2, 4 and 12 h after vancomycin treatment in each group (μg/ml)

Group	0.5 h	2 h	4 h	12 h
Control	0	0	0	0
SV	10.21 ± 1.79	3.32 ± 0.58	0.48 ± 0.03	**#**
VP	2.68 ± 0.32	0.71 ± 0.08	0.50 ± 0.04	**#**
SV&VP	14.14 ± 1.18	7.49 ± 0.62	1.40 ± 0.23	**#**

Control (no antibiotics); *SV* Systemic vancomycin (intraperitoneal injection, 88 mg/kg, every 12 h), *VP* Intra-wound vancomycin powder (before closure of the capsule, 88 mg/kg, single dosage), *SV&VP* Systemic vancomycin & intra-wound VP (intraperitoneal injection, 88 mg/kg, every 12 h; combined with intra-wound VP before closure of the capsule, 88 mg/kg, single dosage). *n* = 6. **#** Below the limit of detection (0.1 μg/ml)

## Discussion

Vancomycin is usually used in the treatment MRSA infections by inhibiting cell wall synthesis in Gram-positive bacteria. The traditional post-revision surgery administration of MRSA PJI relies on the effects of intravenous systemic vancomycin and follow by long-term oral antibiotics. The MBEC of vancomycin required to eliminate the MRSA biofilms of the prosthesis and infected tissue was up to 10^2^ to 10^4^ times of the MIC [34, 35]. Obviously, systemic vancomycin could hardly meet the requirement of eradicating MRSA infection in a short period [9]. In recent years, orthopedic surgeons tried to apply intra-articular vancomycin (injection or powder), or/and systemic vancomycin, to control the clinical MRSA infection after one-stage exchange arthroplasty [36–39]. However, these studies are essentially empirical retrospective case reports from a single orthopedic center. The theory of local high concentrations of antibiotics is that antibiotics may permeate into the surgical sites of seroma, hematoma and ischemic tissues that may be inaccessible by systemic antibiotics [9, 40]. Compared with intra-articular injection of vancomycin, intra-wound VP did not require long-term injection of catheters or drainage tube. Besides, daily post-operative intra-articular injections for 4–6 weeks or even longer which was reported in the available literature [37, 41] are unlikely to be implemented in clinical practice because of the fear of secondary infection or complications of the catheter incision through the injection channel. Thus, intra-articular catheter injection of vancomycin seems not to be the best way to achieve the MBEC in the synovial fluid and infected tissues after one-stage exchange arthroplasty. Although our results indicated that intra-wound VP alone could not eliminate the bacteria, it might be an important complement to systemic administration. When systemic vancomycin in combination with intra-wound VP could eradicate MRSA infection within a short period as confirmed by our current study. Our data suggested that systemic vancomycin plus intra-wound VP might be an effective way to eliminate MRSA infection after one-stage exchange arthroplasty in 2 weeks in our current rat PJI model.

The most commonly reported adverse effects of vancomycin used in human are nephrotoxicity and ototoxicity. Clinical nephrotoxicity of routine systemic vancomycin which have been reported ranged from 0 to 17% [42–44]. In this study, no adverse effects of vancomycin were observed in serum biochemistry between the Control group and vancomycin therapeutic groups. Meanwhile, our data also indicated that the serum levels of vancomycin in the treatment groups were all higher than the MIC of MRSA at 0.5 h after administration, but lower than the serum level that could cause nephrotoxicity. Another concern is the osteoblasts toxicity caused by local high concentrations of vancomycin, studies have showed that high dosage of antibiotics such as fluoroquinolone in surgical area could delay bone healing and repairing, with vancomycin being less toxic than ciprofloxacin or tobramycin [45]. On the base of the previous literature, if the concentration of vancomycin was less than 1 mg/ml, the toxicity to osteoblasts growth and activity was minimum at the cellular level [46–48]. The available literature indicated that osteolytic damage was not observed in the intra-wound application of VP. Besides, the introduction of a crystalline substance (such as vancomycin powder) into the prosthetic interface caused concern about third-body wear. However, an in vitro mechanical study had demonstrated no acceleration in the prosthetic interface wear by VP [49]. Furthermore, one concern is the seroma formation with the local vancomycin treatment, a clinical study suggested that there was no significant difference in seroma formation between the control group and intra-articular VP group [15]. Although the local or systemic adverse effects of vancomycin were not observed in our study, the potential effects still existed. Further investigations and summary of drug-toxicity and the effect on metal-polyethylene wear in future clinical applications remain necessary.

However, there are still some limitations in this study. Firstly, our data were based on the uncemented prosthesis, which might lead to the prosthesis loosening and osteolysis around the prosthesis on the early stage after surgery. Thus, the prosthesis loosening and osteolysis around the prosthesis by the micromovement of the prosthesis itself might accelerate the osteolysis caused by the bacteria alone. Further studies are needed to explore the prosthesis loosening and osteolysis by using the cemented prosthesis. Secondly, the absorption, distribution, metabolism and excretion of drugs in the murine is not the same as the humans, the biodynamics and biomechanics of the rat knee is also not the same as human’s, which means the current rat-based study could not exactly mimic the PJI occurred in the patients with arthroplasty surgery. Further clinical observation and study are needed to maintain more direct evidence of the effectiveness and safety of the VP in the one-stage exchange surgery. Thirdly, the 14-day systemic vancomycin treatment post-exchange surgery may be inadequate, but the elimination of MRSA infection had been observed for this duration in the SV&VP group. Lastly, our study focused on the efficacy of intra-wound VP after debridement and implant exchange, without including adjuvant oral antibiotics, such as rifampin. Further studies are needed in the future.

In conclusion, in the current rat PJI model, intra-wound vancomycin powder is effective and safe to control the infection after debridement and implant exchange, while the combination of systemic vancomycin and intra-wound VP can completely eradicate the MRSA infection. Our experiment data support the potential clinical application of systemic vancomycin plus intra-wound vancomycin powder in the one-stage revision surgery. More clinical trials and follow-ups are needed in the future.

## Data Availability

All relevant data analyzed during the current study are within the paper.

## Abbreviations

VP: Vancomycin powder
PJI: Periprosthetic joint infection
MRSA: Methicillin-resistant *S. aureus*
MBEC: Minimum biofilm eradication concentration
MIC: Minimal inhibitory concentration
α1-AGP: Alpha-1-acid glycoprotein
Cr: Creatinine
ALT: Alanine aminotransferase
AST: Aspartate aminotransferase
HPLC-MS: High-performance liquid chromatography-mass spectrometry
SEM: Scanning electron microscopy
H&E: Hematoxylin and eosin
